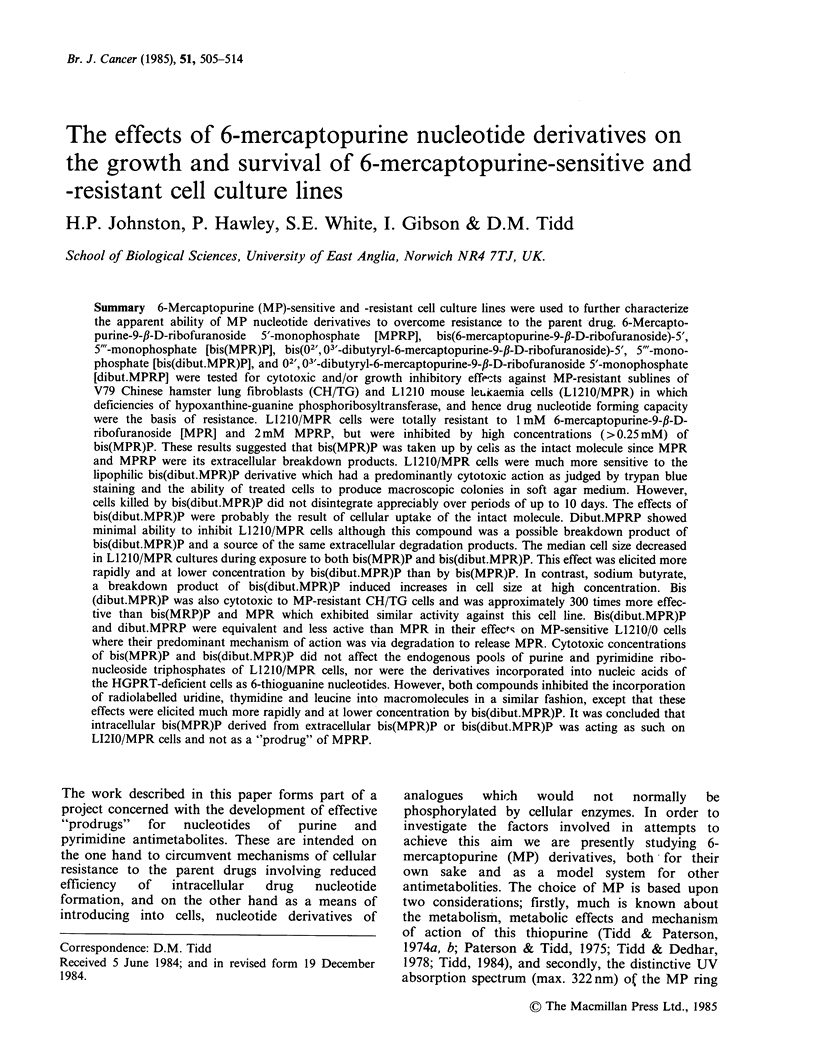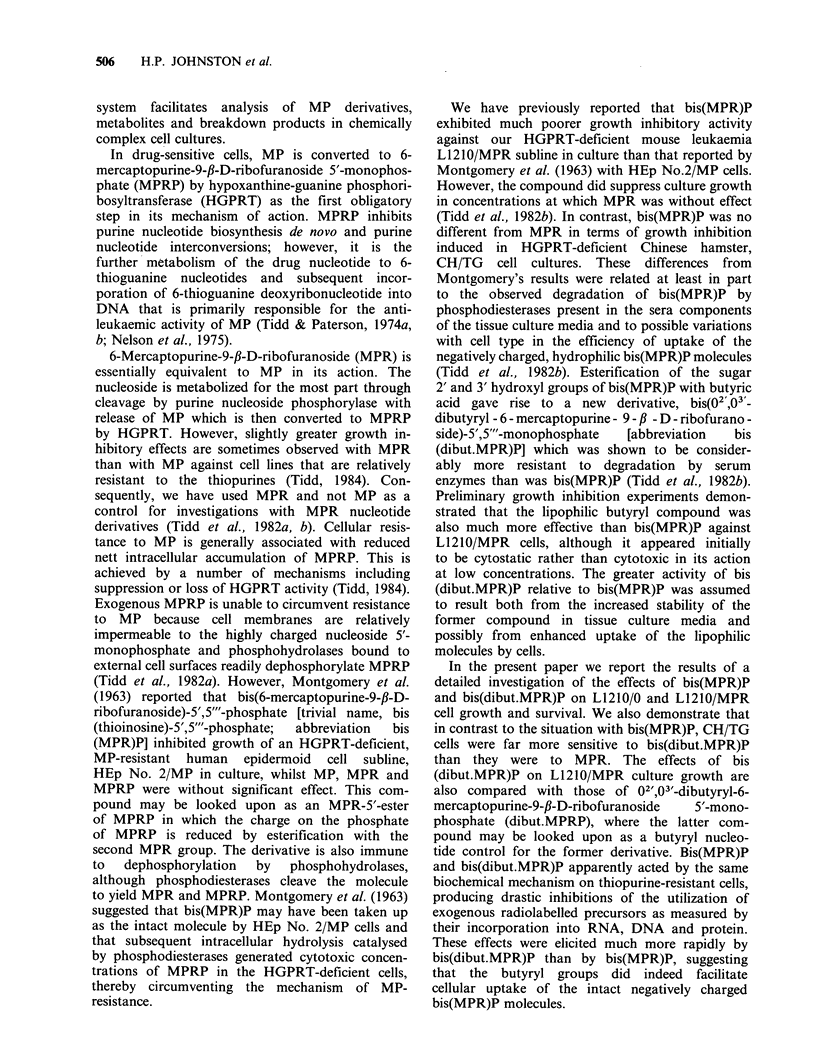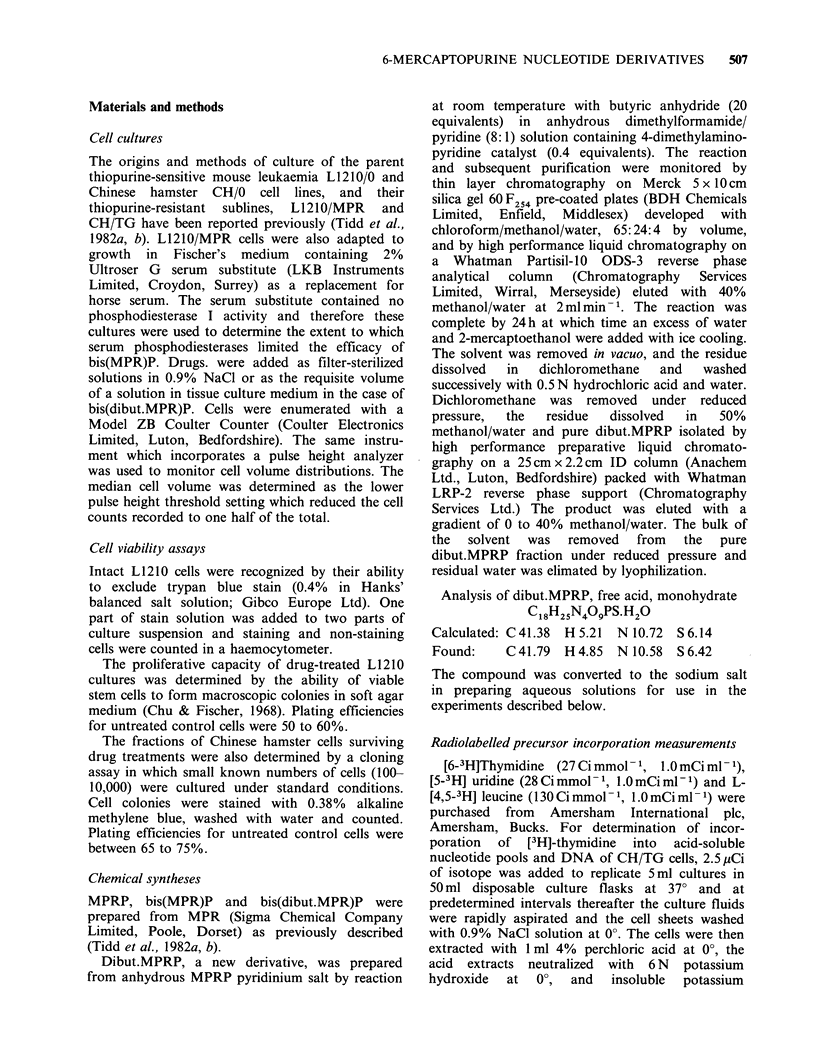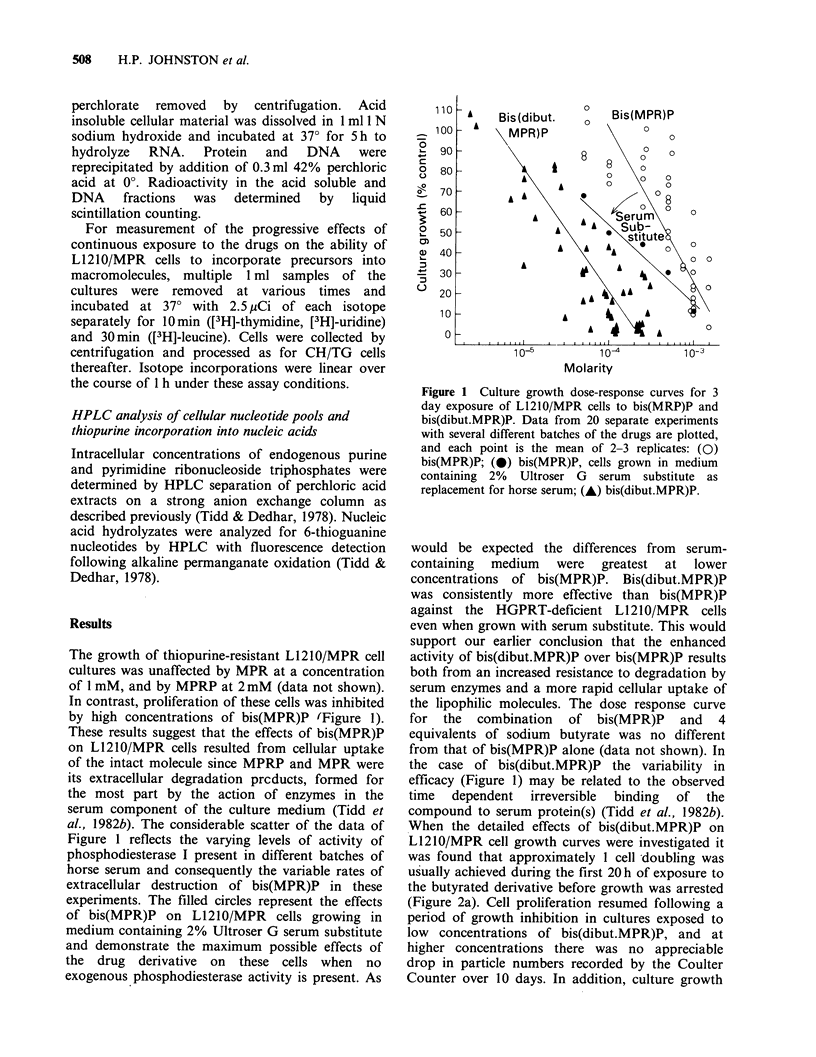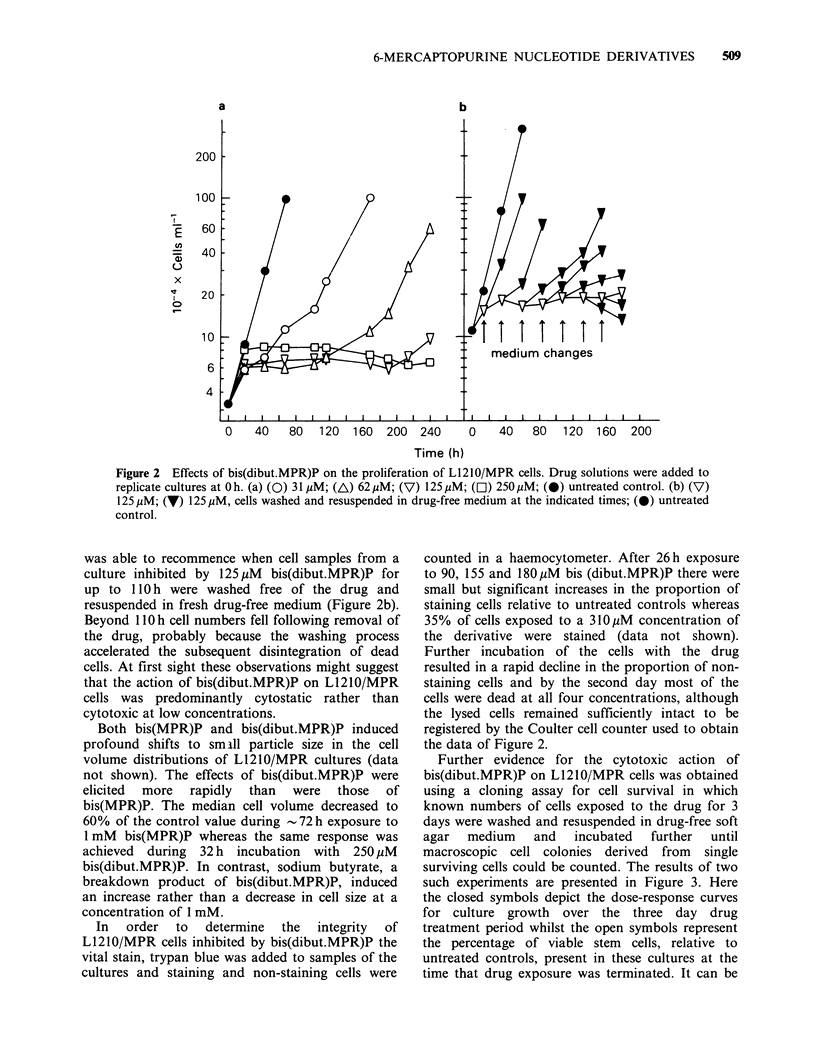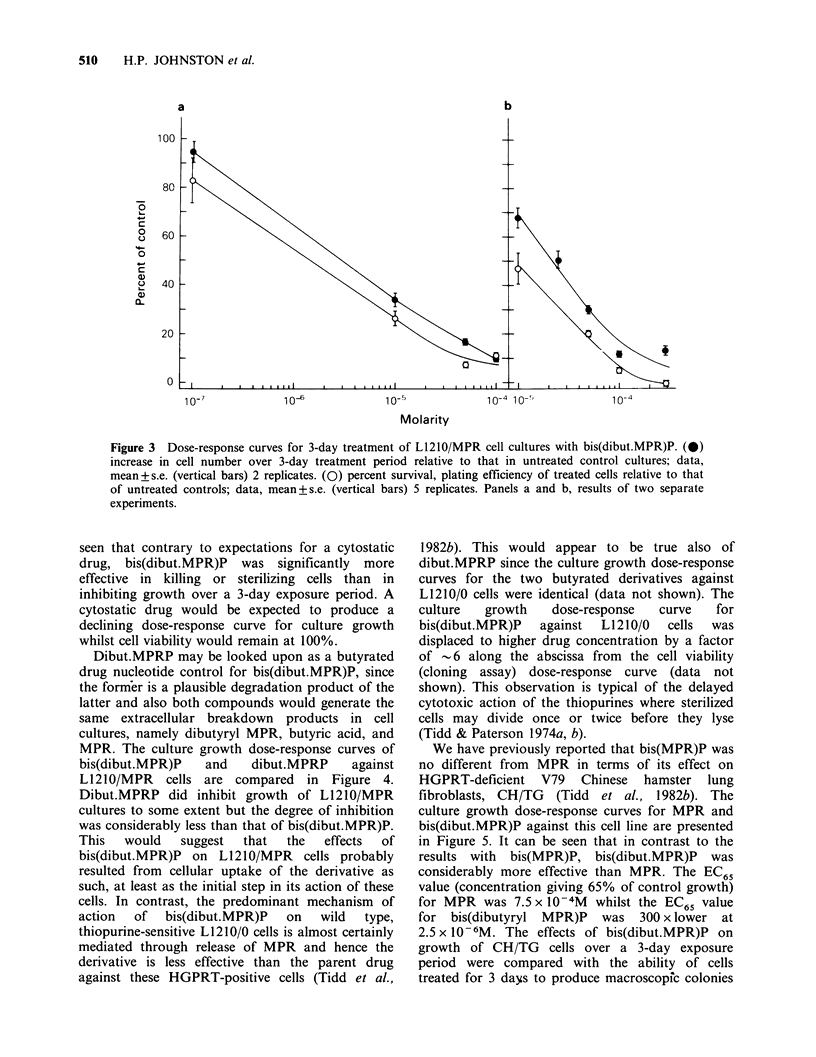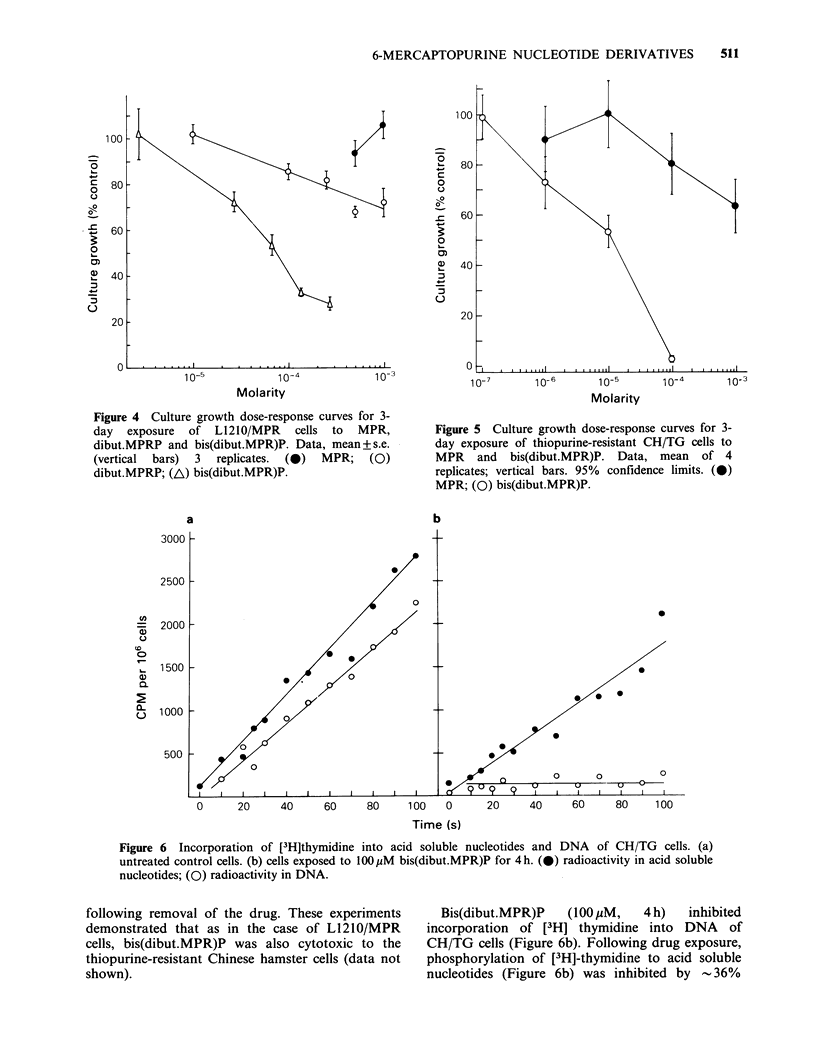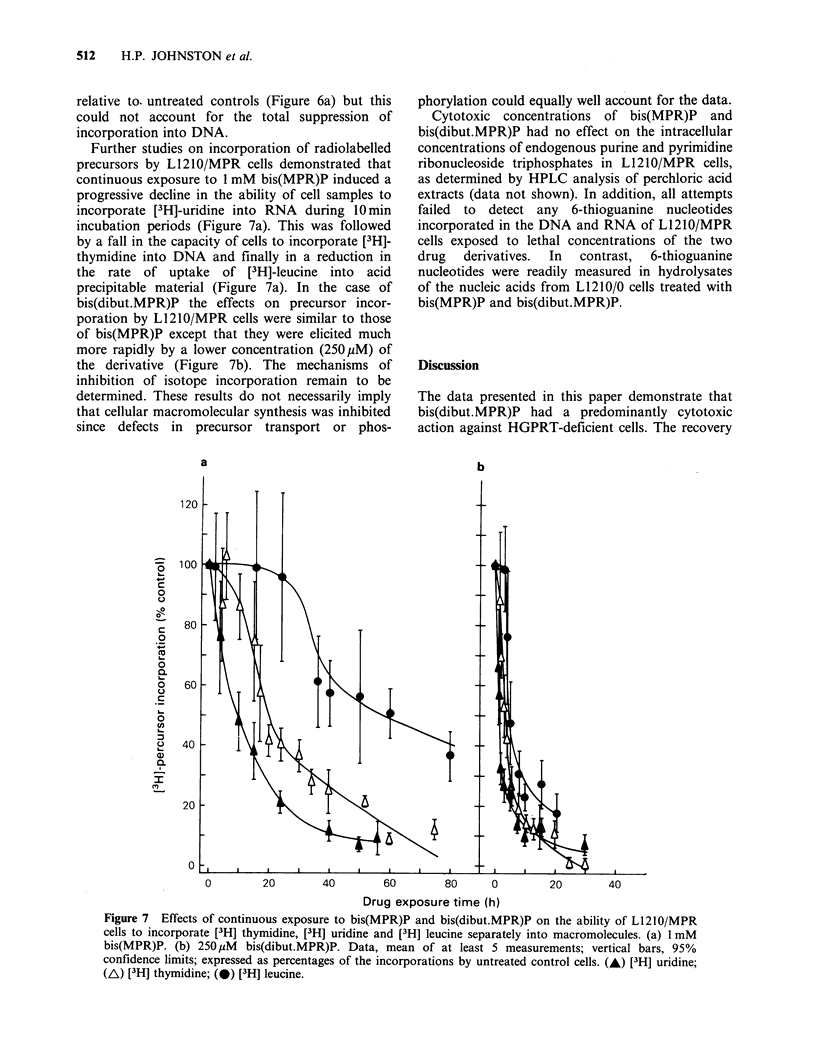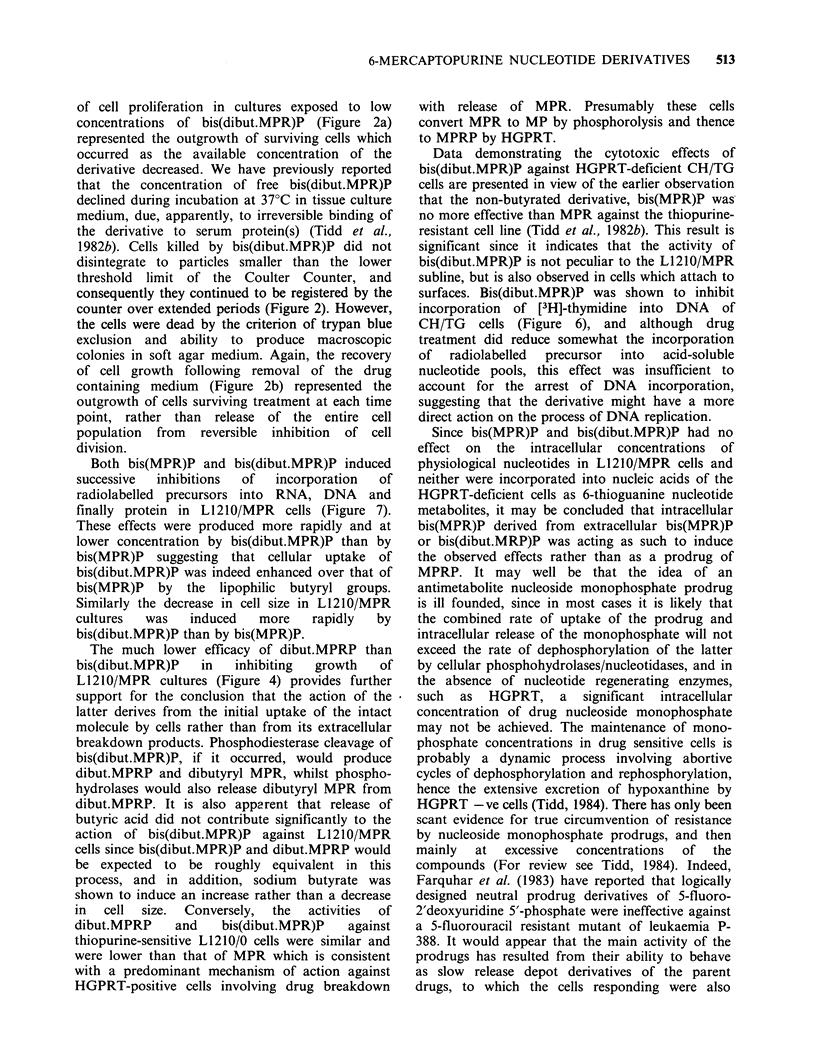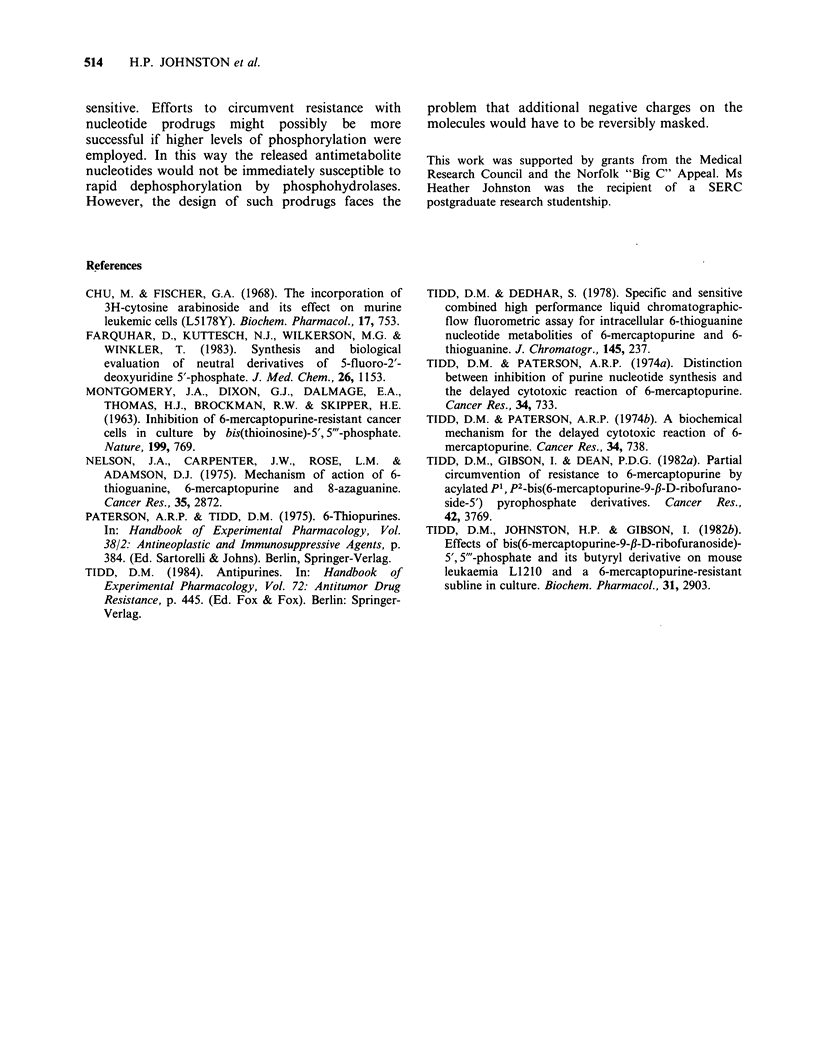# The effects of 6-mercaptopurine nucleotide derivatives on the growth and survival of 6-mercaptopurine-sensitive and -resistant cell culture lines.

**DOI:** 10.1038/bjc.1985.73

**Published:** 1985-04

**Authors:** H. P. Johnston, P. Hawley, S. E. White, I. Gibson, D. M. Tidd

## Abstract

6-Mercaptopurine (MP)-sensitive and -resistant cell culture lines were used to further characterize the apparent ability of MP nucleotide derivatives to overcome resistance to the parent drug. 6-Mercaptopurine-9-beta-D-ribofuranoside 5'-monophosphate [MPRP], bis(6-mercaptopurine-9-beta-D-ribofuranoside)-5', 5"'-monophosphate [bis(MPR)P], bis(O2',O3'-dibutyryl-6-mercaptopurine-9-beta-D-ribofuranoside)-5', 5"'-monophosphate [bis(dibut.MPR)P], and O2',O3'-dibutyryl-6-mercaptopurine-9-beta-D-ribofuranoside 5'-monophosphate [dibut.MPRP] were tested for cytotoxic and/or growth inhibitory effects against MP-resistant sublines of V79 Chinese hamster lung fibroblasts (CH/TG) and L1210 mouse leukaemia cells (L1210/MPR) in which deficiencies of hypoxanthine-guanine phosphoribosyltransferase, and hence drug nucleotide forming capacity were the basis of resistance. L1210/MPR cells were totally resistant to 1 mM 6-mercaptopurine-9-beta-D-ribofuranoside [MPR] and 2 mM MPRP, but were inhibited by high concentrations (greater than 0.25 mM) of bis(MPR)P. These results suggested that bis(MPR)P was taken up by cells as the intact molecule since MPR and MPRP were its extracellular breakdown products. L1210/MPR cells were much more sensitive to the lipophilic bis(dibut.MPR)P derivative which had a predominantly cytotoxic action as judged by trypan blue staining and the ability of treated cells to produce macroscopic colonies in soft agar medium. However, cells killed by bis(dibut.MPR)P did not disintegrate appreciably over periods of up to 10 days. The effects of bis(dibut.MPR)P were probably the result of cellular uptake of the intact molecule. Dibut.MPRP showed minimal ability to inhibit L1210/MPR cells although this compound was a possible breakdown product of bis(dibut.MPR)P and a source of the same extracellular degradation products. The median cell size decreased in L1210/MPR cultures during exposure to both bis(MPR)P and bis(dibut.MPR)P. This effect was elicited more rapidly and at lower concentration by bis(dibut.MPR)P than by bis(MPR)P. In contrast, sodium butyrate, a breakdown product of bis(dibut.MPR)P induced increases in cell size at high concentration. Bis (dibut.MPR)P was also cytotoxic to MP-resistant CH/TG cells and was approximately 300 times more effective than bis(MRP)P and MPR which exhibited similar activity against this cell line. Bis(dibut.MPR)P and dibut.MPRP were equivalent and less active than MPR in their effects on MP-sensitive L1210/0 cells where their predominant mechanism of action was via degradation to release MPR.(ABSTRACT TRUNCATED AT 400 WORDS)


*Br. J. Cancer* (1985), **51**, 505–514

### The effects of 6-mercaptopurine nucleotide derivatives on the growth and survival of 6-mercaptopurine-sensitive and -resistant cell culture lines

H.P. Johnston, P. Hawley, S.E. White, I. Gibson & D.M. Tidd

*School of Biological Sciences, University of East Anglia, Norwich NR4 7TJ, UK.*

**Summary** 6-Mercaptopurine (MP)-sensitive and -resistant cell culture lines were used to further characterize the apparent ability of MP nucleotide derivatives to overcome resistance to the parent drug. 6-Mercaptopurine-9-β-D-ribofuranoside 5′-monophosphate [MPRP], bis(6-mercaptopurine-9-β-D-ribofuranoside)-5′, 5‴-monophosphate [bis(MPR)P], bis(0$^{2'}$,0$^{3'}$-dibutyryl-6-mercaptopurine-9-β-D-ribofuranoside)-5′, 5‴-monophosphate [bis(dibut.MPR)P], and 0$^{2'}$,0$^{3'}$-dibutyryl-6-mercaptopurine-9-β-D-ribofuranoside 5′-monophosphate [dibut.MPRP] were tested for cytotoxic and/or growth inhibitory effects against MP-resistant sublines of V79 Chinese hamster lung fibroblasts (CH/TG) and L1210 mouse leukaemia cells (L1210/MPR) in which deficiencies of hypoxanthine-guanine phosphoribosyltransferase, and hence drug nucleotide forming capacity were the basis of resistance. L1210/MPR cells were totally resistant to 1 mM 6-mercaptopurine-9-β-D-ribofuranoside [MPR] and 2 mM MPRP, but were inhibited by high concentrations (>0.25 mM) of bis(MPR)P. These results suggested that bis(MPR)P was taken up by cells as the intact molecule since MPR and MPRP were its extracellular breakdown products. L1210/MPR cells were much more sensitive to the lipophilic bis(dibut.MPR)P derivative which had a predominantly cytotoxic action as judged by trypan blue staining and the ability of treated cells to produce macroscopic colonies in soft agar medium. However, cells killed by bis(dibut.MPR)P did not disintegrate appreciably over periods of up to 10 days. The effects of bis(dibut.MPR)P were probably the result of cellular uptake of the intact molecule. Dibut.MPRP showed minimal ability to inhibit L1210/MPR cells although this compound was a possible breakdown product of bis(dibut.MPR)P and a source of the same extracellular degradation products. The median cell size decreased in L1210/MPR cultures during exposure to both bis(MPR)P and bis(dibut.MPR)P. This effect was elicited more rapidly and at lower concentration by bis(dibut.MPR)P than by bis(MPR)P. In contrast, sodium butyrate, a breakdown product of bis(dibut.MPR)P induced increases in cell size at high concentration. Bis (dibut.MPR)P was also cytotoxic to MP-resistant CH/TG cells and was approximately 300 times more effective than bis(MRP)P and MPR which exhibited similar activity against this cell line. Bis(dibut.MPR)P and dibut.MPRP were equivalent and less active than MPR in their effects on MP-sensitive L1210/0 cells where their predominant mechanism of action was via degradation to release MPR. Cytotoxic concentrations of bis(MPR)P and bis(dibut.MPR)P did not affect the endogenous pools of purine and pyrimidine ribonucleoside triphosphates of L1210/MPR cells, nor were the derivatives incorporated into nucleic acids of the HGPRT-deficient cells as 6-thioguanine nucleotides. However, both compounds inhibited the incorporation of radiolabelled uridine, thymidine and leucine into macromolecules in a similar fashion, except that these effects were elicited much more rapidly and at lower concentration by bis(dibut.MPR)P. It was concluded that intracellular bis(MPR)P derived from extracellular bis(MPR)P or bis(dibut.MPR)P was acting as such on LI2I0/MPR cells and not as a "prodrug" of MPRP.

The work described in this paper forms part of a project concerned with the development of effective "prodrugs" for nucleotides of purine and pyrimidine antimetabolites. These are intended on the one hand to circumvent mechanisms of cellular resistance to the parent drugs involving reduced efficiency of intracellular drug nucleotide formation, and on the other hand as a means of introducing into cells, nucleotide derivatives of analogues which would not normally be phosphorylated by cellular enzymes. In order to investigate the factors involved in attempts to achieve this aim we are presently studying 6-mercaptopurine (MP) derivatives, both for their own sake and as a model system for other antimetabolities. The choice of MP is based upon two considerations; firstly, much is known about the metabolism, metabolic effects and mechanism of action of this thiopurine (Tidd & Paterson, 1974*a*, *b*; Paterson & Tidd, 1975; Tidd & Dedhar, 1978; Tidd, 1984), and secondly, the distinctive UV absorption spectrum (max. 322 nm) of the MP ring

Correspondence: D.M. Tidd
Received 5 June 1984; and in revised form 19 December 1984.

© The Macmillan Press Ltd., 1985

system facilitates analysis of MP derivatives, metabolites and breakdown products in chemically complex cell cultures.

In drug-sensitive cells, MP is converted to 6-mercaptopurine-9-β-D-ribofuranoside 5′-monophosphate (MPRP) by hypoxanthine-guanine phosphoribosyltransferase (HGPRT) as the first obligatory step in its mechanism of action. MPRP inhibits purine nucleotide biosynthesis *de novo* and purine nucleotide interconversions; however, it is the further metabolism of the drug nucleotide to 6-thioguanine nucleotides and subsequent incorporation of 6-thioguanine deoxyribonucleotide into DNA that is primarily responsible for the anti-leukaemic activity of MP (Tidd & Paterson, 1974*a*, *b*; Nelson *et al.*, 1975).

6-Mercaptopurine-9-β-D-ribofuranoside (MPR) is essentially equivalent to MP in its action. The nucleoside is metabolized for the most part through cleavage by purine nucleoside phosphorylase with release of MP which is then converted to MPRP by HGPRT. However, slightly greater growth inhibitory effects are sometimes observed with MPR than with MP against cell lines that are relatively resistant to the thiopurines (Tidd, 1984). Consequently, we have used MPR and not MP as a control for investigations with MPR nucleotide derivatives (Tidd *et al.*, 1982*a*, *b*). Cellular resistance to MP is generally associated with reduced nett intracellular accumulation of MPRP. This is achieved by a number of mechanisms including suppression or loss of HGPRT activity (Tidd, 1984). Exogenous MPRP is unable to circumvent resistance to MP because cell membranes are relatively impermeable to the highly charged nucleoside 5′-monophosphate and phosphohydrolases bound to external cell surfaces readily dephosphorylate MPRP (Tidd *et al.*, 1982*a*). However, Montgomery *et al.* (1963) reported that bis(6-mercaptopurine-9-β-D-ribofuranoside)-5′,5‴-phosphate [trivial name, bis (thioinosine)-5′,5‴-phosphate; abbreviation bis (MPR)P] inhibited growth of an HGPRT-deficient, MP-resistant human epidermoid cell subline, HEp No. 2/MP in culture, whilst MP, MPR and MPRP were without significant effect. This compound may be looked upon as an MPR-5′-ester of MPRP in which the charge on the phosphate of MPRP is reduced by esterification with the second MPR group. The derivative is also immune to dephosphorylation by phosphohydrolases, although phosphodiesterases cleave the molecule to yield MPR and MPRP. Montgomery *et al.* (1963) suggested that bis(MPR)P may have been taken up as the intact molecule by HEp No. 2/MP cells and that subsequent intracellular hydrolysis catalysed by phosphodiesterases generated cytotoxic concentrations of MPRP in the HGPRT-deficient cells, thereby circumventing the mechanism of MP-resistance.

We have previously reported that bis(MPR)P exhibited much poorer growth inhibitory activity against our HGPRT-deficient mouse leukaemia L1210/MPR subline in culture than that reported by Montgomery *et al.* (1963) with HEp No.2/MP cells. However, the compound did suppress culture growth in concentrations at which MPR was without effect (Tidd *et al.*, 1982*b*). In contrast, bis(MPR)P was no different from MPR in terms of growth inhibition induced in HGPRT-deficient Chinese hamster, CH/TG cell cultures. These differences from Montgomery's results were related at least in part to the observed degradation of bis(MPR)P by phosphodiesterases present in the sera components of the tissue culture media and to possible variations with cell type in the efficiency of uptake of the negatively charged, hydrophilic bis(MPR)P molecules (Tidd *et al.*, 1982*b*). Esterification of the sugar 2′ and 3′ hydroxyl groups of bis(MPR)P with butyric acid gave rise to a new derivative, bis($0^{2'}$,$0^{3'}$-dibutyryl-6-mercaptopurine-9-β-D-ribofuranoside)-5′,5‴-monophosphate [abbreviation bis (dibut.MPR)P] which was shown to be considerably more resistant to degradation by serum enzymes than was bis(MPR)P (Tidd *et al.*, 1982*b*). Preliminary growth inhibition experiments demonstrated that the lipophilic butyryl compound was also much more effective than bis(MPR)P against L1210/MPR cells, although it appeared initially to be cytostatic rather than cytotoxic in its action at low concentrations. The greater activity of bis (dibut.MPR)P relative to bis(MPR)P was assumed to result both from the increased stability of the former compound in tissue culture media and possibly from enhanced uptake of the lipophilic molecules by cells.

In the present paper we report the results of a detailed investigation of the effects of bis(MPR)P and bis(dibut.MPR)P on L1210/0 and L1210/MPR cell growth and survival. We also demonstrate that in contrast to the situation with bis(MPR)P, CH/TG cells were far more sensitive to bis(dibut.MPR)P than they were to MPR. The effects of bis (dibut.MPR)P on L1210/MPR culture growth are also compared with those of $0^{2'}$,$0^{3'}$-dibutyryl-6-mercaptopurine-9-β-D-ribofuranoside 5′-monophosphate (dibut.MPRP), where the latter compound may be looked upon as a butyryl nucleotide control for the former derivative. Bis(MPR)P and bis(dibut.MPR)P apparently acted by the same biochemical mechanism on thiopurine-resistant cells, producing drastic inhibitions of the utilization of exogenous radiolabelled precursors as measured by their incorporation into RNA, DNA and protein. These effects were elicited much more rapidly by bis(dibut.MPR)P than by bis(MPR)P, suggesting that the butyryl groups did indeed facilitate cellular uptake of the intact negatively charged bis(MPR)P molecules.

### Materials and methods

### *Cell cultures*

The origins and methods of culture of the parent thiopurine-sensitive mouse leukaemia L1210/0 and Chinese hamster CH/0 cell lines, and their thiopurine-resistant sublines, L1210/MPR and CH/TG have been reported previously (Tidd *et al.*, 1982*a*, *b*). L1210/MPR cells were also adapted to growth in Fischer's medium containing 2% Ultroser G serum substitute (LKB Instruments Limited, Croydon, Surrey) as a replacement for horse serum. The serum substitute contained no phosphodiesterase I activity and therefore these cultures were used to determine the extent to which serum phosphodiesterases limited the efficacy of bis(MPR)P. Drugs. were added as filter-sterilized solutions in 0.9% NaCl or as the requisite volume of a solution in tissue culture medium in the case of bis(dibut.MPR)P. Cells were enumerated with a Model ZB Coulter Counter (Coulter Electronics Limited, Luton, Bedfordshire). The same instrument which incorporates a pulse height analyzer was used to monitor cell volume distributions. The median cell volume was determined as the lower pulse height threshold setting which reduced the cell counts recorded to one half of the total.

### *Cell viability assays*

Intact L1210 cells were recognized by their ability to exclude trypan blue stain (0.4% in Hanks' balanced salt solution; Gibco Europe Ltd). One part of stain solution was added to two parts of culture suspension and staining and non-staining cells were counted in a haemocytometer.

The proliferative capacity of drug-treated L1210 cultures was determined by the ability of viable stem cells to form macroscopic colonies in soft agar medium (Chu & Fischer, 1968). Plating efficiencies for untreated control cells were 50 to 60%.

The fractions of Chinese hamster cells surviving drug treatments were also determined by a cloning assay in which small known numbers of cells (100–10,000) were cultured under standard conditions. Cell colonies were stained with 0.38% alkaline methylene blue, washed with water and counted. Plating efficiencies for untreated control cells were between 65 to 75%.

### *Chemical syntheses*

MPRP, bis(MPR)P and bis(dibut.MPR)P were prepared from MPR (Sigma Chemical Company Limited, Poole, Dorset) as previously described (Tidd *et al.*, 1982*a*, *b*).

Dibut.MPRP, a new derivative, was prepared from anhydrous MPRP pyridinium salt by reaction at room temperature with butyric anhydride (20 equivalents) in anhydrous dimethylformamide/pyridine (8:1) solution containing 4-dimethylaminopyridine catalyst (0.4 equivalents). The reaction and subsequent purification were monitored by thin layer chromatography on Merck 5×10 cm silica gel 60 $F_{254}$ pre-coated plates (BDH Chemicals Limited, Enfield, Middlesex) developed with chloroform/methanol/water, 65:24:4 by volume, and by high performance liquid chromatography on a Whatman Partisil-10 ODS-3 reverse phase analytical column (Chromatography Services Limited, Wirral, Merseyside) eluted with 40% methanol/water at 2 ml min$^{-1}$. The reaction was complete by 24 h at which time an excess of water and 2-mercaptoethanol were added with ice cooling. The solvent was removed *in vacuo*, and the residue dissolved in dichloromethane and washed successively with 0.5 N hydrochloric acid and water. Dichloromethane was removed under reduced pressure, the residue dissolved in 50% methanol/water and pure dibut.MPRP isolated by high performance preparative liquid chromatography on a 25 cm × 2.2 cm ID column (Anachem Ltd., Luton, Bedfordshire) packed with Whatman LRP-2 reverse phase support (Chromatography Services Ltd.) The product was eluted with a gradient of 0 to 40% methanol/water. The bulk of the solvent was removed from the pure dibut.MPRP fraction under reduced pressure and residual water was elimated by lyophilization.

Analysis of dibut.MPRP, free acid, monohydrate
$C_{18}H_{25}N_4O_9PS.H_2O$

| | | | | |
|---|---|---|---|---|
| Calculated: | C 41.38 | H 5.21 | N 10.72 | S 6.14 |
| Found: | C 41.79 | H 4.85 | N 10.58 | S 6.42 |

The compound was converted to the sodium salt in preparing aqueous solutions for use in the experiments described below.

### *Radiolabelled precursor incorporation measurements*

[6-$^3$H]Thymidine (27 Ci mmol$^{-1}$, 1.0 mCi ml$^{-1}$), [5-$^3$H] uridine (28 Ci mmol$^{-1}$, 1.0 mCi ml$^{-1}$) and L-[4,5-$^3$H] leucine (130 Ci mmol$^{-1}$, 1.0 mCi ml$^{-1}$) were purchased from Amersham International plc, Amersham, Bucks. For determination of incorporation of [$^3$H]-thymidine into acid-soluble nucleotide pools and DNA of CH/TG cells, 2.5 $\mu$Ci of isotope was added to replicate 5 ml cultures in 50 ml disposable culture flasks at 37° and at predetermined intervals thereafter the culture fluids were rapidly aspirated and the cell sheets washed with 0.9% NaCl solution at 0°. The cells were then extracted with 1 ml 4% perchloric acid at 0°, the acid extracts neutralized with 6 N potassium hydroxide at 0°, and insoluble potassium perchlorate removed by centrifugation. Acid insoluble cellular material was dissolved in 1 ml 1 N sodium hydroxide and incubated at 37° for 5 h to hydrolyze RNA. Protein and DNA were reprecipitated by addition of 0.3 ml 42% perchloric acid at 0°. Radioactivity in the acid soluble and DNA fractions was determined by liquid scintillation counting.

For measurement of the progressive effects of continuous exposure to the drugs on the ability of L1210/MPR cells to incorporate precursors into macromolecules, multiple 1 ml samples of the cultures were removed at various times and incubated at 37° with 2.5 $\mu$Ci of each isotope separately for 10 min ([$^3$H]-thymidine, [$^3$H]-uridine) and 30 min ([$^3$H]-leucine). Cells were collected by centrifugation and processed as for CH/TG cells thereafter. Isotope incorporations were linear over the course of 1 h under these assay conditions.

*HPLC analysis of cellular nucleotide pools and thiopurine incorporation into nucleic acids*

Intracellular concentrations of endogenous purine and pyrimidine ribonucleoside triphosphates were determined by HPLC separation of perchloric acid extracts on a strong anion exchange column as described previously (Tidd & Dedhar, 1978). Nucleic acid hydrolyzates were analyzed for 6-thioguanine nucleotides by HPLC with fluorescence detection following alkaline permanganate oxidation (Tidd & Dedhar, 1978).

### Results

The growth of thiopurine-resistant L1210/MPR cell cultures was unaffected by MPR at a concentration of 1 mM, and by MPRP at 2 mM (data not shown). In contrast, proliferation of these cells was inhibited by high concentrations of bis(MPR)P (Figure 1). These results suggest that the effects of bis(MPR)P on L1210/MPR cells resulted from cellular uptake of the intact molecule since MPRP and MPR were its extracellular degradation products, formed for the most part by the action of enzymes in the serum component of the culture medium (Tidd *et al.*, 1982*b*). The considerable scatter of the data of Figure 1 reflects the varying levels of activity of phosphodiesterase I present in different batches of horse serum and consequently the variable rates of extracellular destruction of bis(MPR)P in these experiments. The filled circles represent the effects of bis(MPR)P on L1210/MPR cells growing in medium containing 2% Ultroser G serum substitute and demonstrate the maximum possible effects of the drug derivative on these cells when no exogenous phosphodiesterase activity is present. As

**Figure 1** Culture growth dose-response curves for 3 day exposure of L1210/MPR cells to bis(MRP)P and bis(dibut.MPR)P. Data from 20 separate experiments with several different batches of the drugs are plotted, and each point is the mean of 2–3 replicates: (○) bis(MPR)P; (●) bis(MPR)P, cells grown in medium containing 2% Ultroser G serum substitute as replacement for horse serum; (▲) bis(dibut.MPR)P.

would be expected the differences from serum-containing medium were greatest at lower concentrations of bis(MPR)P. Bis(dibut.MPR)P was consistently more effective than bis(MPR)P against the HGPRT-deficient L1210/MPR cells even when grown with serum substitute. This would support our earlier conclusion that the enhanced activity of bis(dibut.MPR)P over bis(MPR)P results both from an increased resistance to degradation by serum enzymes and a more rapid cellular uptake of the lipophilic molecules. The dose response curve for the combination of bis(MPR)P and 4 equivalents of sodium butyrate was no different from that of bis(MPR)P alone (data not shown). In the case of bis(dibut.MPR)P the variability in efficacy (Figure 1) may be related to the observed time dependent irreversible binding of the compound to serum protein(s) (Tidd *et al.*, 1982*b*). When the detailed effects of bis(dibut.MPR)P on L1210/MPR cell growth curves were investigated it was found that approximately 1 cell doubling was usually achieved during the first 20 h of exposure to the butyrated derivative before growth was arrested (Figure 2a). Cell proliferation resumed following a period of growth inhibition in cultures exposed to low concentrations of bis(dibut.MPR)P, and at higher concentrations there was no appreciable drop in particle numbers recorded by the Coulter Counter over 10 days. In addition, culture growth

**Figure 2** Effects of bis(dibut.MPR)P on the proliferation of L1210/MPR cells. Drug solutions were added to replicate cultures at 0 h. (a) (○) 31 μM; (△) 62 μM; (▽) 125 μM; (□) 250 μM; (●) untreated control. (b) (▽) 125 μM; (▼) 125 μM, cells washed and resuspended in drug-free medium at the indicated times; (●) untreated control.

was able to recommence when cell samples from a culture inhibited by 125 μM bis(dibut.MPR)P for up to 110 h were washed free of the drug and resuspended in fresh drug-free medium (Figure 2b). Beyond 110 h cell numbers fell following removal of the drug, probably because the washing process accelerated the subsequent disintegration of dead cells. At first sight these observations might suggest that the action of bis(dibut.MPR)P on L1210/MPR cells was predominantly cytostatic rather than cytotoxic at low concentrations.

Both bis(MPR)P and bis(dibut.MPR)P induced profound shifts to small particle size in the cell volume distributions of L1210/MPR cultures (data not shown). The effects of bis(dibut.MPR)P were elicited more rapidly than were those of bis(MPR)P. The median cell volume decreased to 60% of the control value during ~72 h exposure to 1 mM bis(MPR)P whereas the same response was achieved during 32 h incubation with 250 μM bis(dibut.MPR)P. In contrast, sodium butyrate, a breakdown product of bis(dibut.MPR)P, induced an increase rather than a decrease in cell size at a concentration of 1 mM.

In order to determine the integrity of L1210/MPR cells inhibited by bis(dibut.MPR)P the vital stain, trypan blue was added to samples of the cultures and staining and non-staining cells were counted in a haemocytometer. After 26 h exposure to 90, 155 and 180 μM bis (dibut.MPR)P there were small but significant increases in the proportion of staining cells relative to untreated controls whereas 35% of cells exposed to a 310 μM concentration of the derivative were stained (data not shown). Further incubation of the cells with the drug resulted in a rapid decline in the proportion of non-staining cells and by the second day most of the cells were dead at all four concentrations, although the lysed cells remained sufficiently intact to be registered by the Coulter cell counter used to obtain the data of Figure 2.

Further evidence for the cytotoxic action of bis(dibut.MPR)P on L1210/MPR cells was obtained using a cloning assay for cell survival in which known numbers of cells exposed to the drug for 3 days were washed and resuspended in drug-free soft agar medium and incubated further until macroscopic cell colonies derived from single surviving cells could be counted. The results of two such experiments are presented in Figure 3. Here the closed symbols depict the dose-response curves for culture growth over the three day drug treatment period whilst the open symbols represent the percentage of viable stem cells, relative to untreated controls, present in these cultures at the time that drug exposure was terminated. It can be

**Figure 3** Dose-response curves for 3-day treatment of L1210/MPR cell cultures with bis(dibut.MPR)P. (●) increase in cell number over 3-day treatment period relative to that in untreated control cultures; data, mean ± s.e. (vertical bars) 2 replicates. (○) percent survival, plating efficiency of treated cells relative to that of untreated controls; data, mean ± s.e. (vertical bars) 5 replicates. Panels a and b, results of two separate experiments.

seen that contrary to expectations for a cytostatic drug, bis(dibut.MPR)P was significantly more effective in killing or sterilizing cells than in inhibiting growth over a 3-day exposure period. A cytostatic drug would be expected to produce a declining dose-response curve for culture growth whilst cell viability would remain at 100%.

Dibut.MPRP may be looked upon as a butyrated drug nucleotide control for bis(dibut.MPR)P, since the former is a plausible degradation product of the latter and also both compounds would generate the same extracellular breakdown products in cell cultures, namely dibutyryl MPR, butyric acid, and MPR. The culture growth dose-response curves of bis(dibut.MPR)P and dibut.MPRP against L1210/MPR cells are compared in Figure 4. Dibut.MPRP did inhibit growth of L1210/MPR cultures to some extent but the degree of inhibition was considerably less than that of bis(dibut.MPR)P. This would suggest that the effects of bis(dibut.MPR)P on L1210/MPR cells probably resulted from cellular uptake of the derivative as such, at least as the initial step in its action of these cells. In contrast, the predominant mechanism of action of bis(dibut.MPR)P on wild type, thiopurine-sensitive L1210/0 cells is almost certainly mediated through release of MPR and hence the derivative is less effective than the parent drug against these HGPRT-positive cells (Tidd et al., 1982b). This would appear to be true also of dibut.MPRP since the culture growth dose-response curves for the two butyrated derivatives against L1210/0 cells were identical (data not shown). The culture growth dose-response curve for bis(dibut.MPR)P against L1210/0 cells was displaced to higher drug concentration by a factor of ~6 along the abscissa from the cell viability (cloning assay) dose-response curve (data not shown). This observation is typical of the delayed cytotoxic action of the thiopurines where sterilized cells may divide once or twice before they lyse (Tidd & Paterson 1974a, b).

We have previously reported that bis(MPR)P was no different from MPR in terms of its effect on HGPRT-deficient V79 Chinese hamster lung fibroblasts, CH/TG (Tidd et al., 1982b). The culture growth dose-response curves for MPR and bis(dibut.MPR)P against this cell line are presented in Figure 5. It can be seen that in contrast to the results with bis(MPR)P, bis(dibut.MPR)P was considerably more effective than MPR. The $EC_{65}$ value (concentration giving 65% of control growth) for MPR was $7.5 \times 10^{-4}$M whilst the $EC_{65}$ value for bis(dibutyryl MPR)P was 300 × lower at $2.5 \times 10^{-6}$M. The effects of bis(dibut.MPR)P on growth of CH/TG cells over a 3-day exposure period were compared with the ability of cells treated for 3 days to produce macroscopic colonies

**Figure 4** Culture growth dose-response curves for 3-day exposure of L1210/MPR cells to MPR, dibut.MPRP and bis(dibut.MPR)P. Data, mean ± s.e. (vertical bars) 3 replicates. (●) MPR; (○) dibut.MPRP; (△) bis(dibut.MPR)P.

**Figure 5** Culture growth dose-response curves for 3-day exposure of thiopurine-resistant CH/TG cells to MPR and bis(dibut.MPR)P. Data, mean of 4 replicates; vertical bars. 95% confidence limits. (●) MPR; (○) bis(dibut.MPR)P.

**Figure 6** Incorporation of [$^3$H]thymidine into acid soluble nucleotides and DNA of CH/TG cells. (a) untreated control cells. (b) cells exposed to 100 $\mu$M bis(dibut.MPR)P for 4 h. (●) radioactivity in acid soluble nucleotides; (○) radioactivity in DNA.

following removal of the drug. These experiments demonstrated that as in the case of L1210/MPR cells, bis(dibut.MPR)P was also cytotoxic to the thiopurine-resistant Chinese hamster cells (data not shown).

Bis(dibut.MPR)P (100 $\mu$M, 4 h) inhibited incorporation of [$^3$H] thymidine into DNA of CH/TG cells (Figure 6b). Following drug exposure, phosphorylation of [$^3$H]-thymidine to acid soluble nucleotides (Figure 6b) was inhibited by ~36%

relative to untreated controls (Figure 6a) but this could not account for the total suppression of incorporation into DNA.

Further studies on incorporation of radiolabelled precursors by L1210/MPR cells demonstrated that continuous exposure to 1 mM bis(MPR)P induced a progressive decline in the ability of cell samples to incorporate [$^3$H]-uridine into RNA during 10 min incubation periods (Figure 7a). This was followed by a fall in the capacity of cells to incorporate [$^3$H]-thymidine into DNA and finally in a reduction in the rate of uptake of [$^3$H]-leucine into acid precipitable material (Figure 7a). In the case of bis(dibut.MPR)P the effects on precursor incorporation by L1210/MPR cells were similar to those of bis(MPR)P except that they were elicited much more rapidly by a lower concentration (250 $\mu$M) of the derivative (Figure 7b). The mechanisms of inhibition of isotope incorporation remain to be determined. These results do not necessarily imply that cellular macromolecular synthesis was inhibited since defects in precursor transport or phosphorylation could equally well account for the data.

Cytotoxic concentrations of bis(MPR)P and bis(dibut.MPR)P had no effect on the intracellular concentrations of endogenous purine and pyrimidine ribonucleoside triphosphates in L1210/MPR cells, as determined by HPLC analysis of perchloric acid extracts (data not shown). In addition, all attempts failed to detect any 6-thioguanine nucleotides incorporated in the DNA and RNA of L1210/MPR cells exposed to lethal concentrations of the two drug derivatives. In contrast, 6-thioguanine nucleotides were readily measured in hydrolysates of the nucleic acids from L1210/0 cells treated with bis(MPR)P and bis(dibut.MPR)P.

### Discussion

The data presented in this paper demonstrate that bis(dibut.MPR)P had a predominantly cytotoxic action against HGPRT-deficient cells. The recovery

**Figure 7** Effects of continuous exposure to bis(MPR)P and bis(dibut.MPR)P on the ability of L1210/MPR cells to incorporate [$^3$H] thymidine, [$^3$H] uridine and [$^3$H] leucine separately into macromolecules. (a) 1 mM bis(MPR)P. (b) 250 $\mu$M bis(dibut.MPR)P. Data, mean of at least 5 measurements; vertical bars, 95% confidence limits; expressed as percentages of the incorporations by untreated control cells. (▲) [$^3$H] uridine; (△) [$^3$H] thymidine; (●) [$^3$H] leucine.

of cell proliferation in cultures exposed to low concentrations of bis(dibut.MPR)P (Figure 2a) represented the outgrowth of surviving cells which occurred as the available concentration of the derivative decreased. We have previously reported that the concentration of free bis(dibut.MPR)P declined during incubation at 37°C in tissue culture medium, due, apparently, to irreversible binding of the derivative to serum protein(s) (Tidd *et al.*, 1982*b*). Cells killed by bis(dibut.MPR)P did not disintegrate to particles smaller than the lower threshold limit of the Coulter Counter, and consequently they continued to be registered by the counter over extended periods (Figure 2). However, the cells were dead by the criterion of trypan blue exclusion and ability to produce macroscopic colonies in soft agar medium. Again, the recovery of cell growth following removal of the drug containing medium (Figure 2b) represented the outgrowth of cells surviving treatment at each time point, rather than release of the entire cell population from reversible inhibition of cell division.

Both bis(MPR)P and bis(dibut.MPR)P induced successive inhibitions of incorporation of radiolabelled precursors into RNA, DNA and finally protein in L1210/MPR cells (Figure 7). These effects were produced more rapidly and at lower concentration by bis(dibut.MPR)P than by bis(MPR)P suggesting that cellular uptake of bis(dibut.MPR)P was indeed enhanced over that of bis(MPR)P by the lipophilic butyryl groups. Similarly the decrease in cell size in L1210/MPR cultures was induced more rapidly by bis(dibut.MPR)P than by bis(MPR)P.

The much lower efficacy of dibut.MPRP than bis(dibut.MPR)P in inhibiting growth of L1210/MPR cultures (Figure 4) provides further support for the conclusion that the action of the latter derives from the initial uptake of the intact molecule by cells rather than from its extracellular breakdown products. Phosphodiesterase cleavage of bis(dibut.MPR)P, if it occurred, would produce dibut.MPRP and dibutyryl MPR, whilst phosphohydrolases would also release dibutyryl MPR from dibut.MPRP. It is also apparent that release of butyric acid did not contribute significantly to the action of bis(dibut.MPR)P against L1210/MPR cells since bis(dibut.MPR)P and dibut.MPRP would be expected to be roughly equivalent in this process, and in addition, sodium butyrate was shown to induce an increase rather than a decrease in cell size. Conversely, the activities of dibut.MPRP and bis(dibut.MPR)P against thiopurine-sensitive L1210/0 cells were similar and were lower than that of MPR which is consistent with a predominant mechanism of action against HGPRT-positive cells involving drug breakdown with release of MPR. Presumably these cells convert MPR to MP by phosphorolysis and thence to MPRP by HGPRT.

Data demonstrating the cytotoxic effects of bis(dibut.MPR)P against HGPRT-deficient CH/TG cells are presented in view of the earlier observation that the non-butyrated derivative, bis(MPR)P was no more effective than MPR against the thiopurine-resistant cell line (Tidd *et al.*, 1982*b*). This result is significant since it indicates that the activity of bis(dibut.MPR)P is not peculiar to the L1210/MPR subline, but is also observed in cells which attach to surfaces. Bis(dibut.MPR)P was shown to inhibit incorporation of [$^{3}$H]-thymidine into DNA of CH/TG cells (Figure 6), and although drug treatment did reduce somewhat the incorporation of radiolabelled precursor into acid-soluble nucleotide pools, this effect was insufficient to account for the arrest of DNA incorporation, suggesting that the derivative might have a more direct action on the process of DNA replication.

Since bis(MPR)P and bis(dibut.MPR)P had no effect on the intracellular concentrations of physiological nucleotides in L1210/MPR cells and neither were incorporated into nucleic acids of the HGPRT-deficient cells as 6-thioguanine nucleotide metabolites, it may be concluded that intracellular bis(MPR)P derived from extracellular bis(MPR)P or bis(dibut.MRP)P was acting as such to induce the observed effects rather than as a prodrug of MPRP. It may well be that the idea of an antimetabolite nucleoside monophosphate prodrug is ill founded, since in most cases it is likely that the combined rate of uptake of the prodrug and intracellular release of the monophosphate will not exceed the rate of dephosphorylation of the latter by cellular phosphohydrolases/nucleotidases, and in the absence of nucleotide regenerating enzymes, such as HGPRT, a significant intracellular concentration of drug nucleoside monophosphate may not be achieved. The maintenance of monophosphate concentrations in drug sensitive cells is probably a dynamic process involving abortive cycles of dephosphorylation and rephosphorylation, hence the extensive excretion of hypoxanthine by HGPRT −ve cells (Tidd, 1984). There has only been scant evidence for true circumvention of resistance by nucleoside monophosphate prodrugs, and then mainly at excessive concentrations of the compounds (For review see Tidd, 1984). Indeed, Farquhar *et al.* (1983) have reported that logically designed neutral prodrug derivatives of 5-fluoro-2′deoxyuridine 5′-phosphate were ineffective against a 5-fluorouracil resistant mutant of leukaemia P-388. It would appear that the main activity of the prodrugs has resulted from their ability to behave as slow release depot derivatives of the parent drugs, to which the cells responding were also sensitive. Efforts to circumvent resistance with nucleotide prodrugs might possibly be more successful if higher levels of phosphorylation were employed. In this way the released antimetabolite nucleotides would not be immediately susceptible to rapid dephosphorylation by phosphohydrolases. However, the design of such prodrugs faces the problem that additional negative charges on the molecules would have to be reversibly masked.

This work was supported by grants from the Medical Research Council and the Norfolk "Big C" Appeal. Ms Heather Johnston was the recipient of a SERC postgraduate research studentship.

### References

CHU, M. & FISCHER, G.A. (1968). The incorporation of 3H-cytosine arabinoside and its effect on murine leukemic cells (L5178Y). *Biochem. Pharmacol.*, **17,** 753.

FARQUHAR, D., KUTTESCH, N.J., WILKERSON, M.G. & WINKLER, T. (1983). Synthesis and biological evaluation of neutral derivatives of 5-fluoro-2′-deoxyuridine 5′-phosphate. *J. Med. Chem.*, **26,** 1153.

MONTGOMERY, J.A., DIXON, G.J., DALMAGE, E.A., THOMAS, H.J., BROCKMAN, R.W. & SKIPPER, H.E. (1963). Inhibition of 6-mercaptopurine-resistant cancer cells in culture by *bis*(thioinosine)-5′,5‴-phosphate. *Nature,* **199,** 769.

NELSON, J.A., CARPENTER, J.W., ROSE, L.M. & ADAMSON, D.J. (1975). Mechanism of action of 6-thioguanine, 6-mercaptopurine and 8-azaguanine. *Cancer Res.*, **35,** 2872.

PATERSON, A.R.P. & TIDD, D.M. (1975). 6-Thiopurines. In: *Handbook of Experimental Pharmacology, Vol. 38/2: Antineoplastic and Immunosuppressive Agents,* p. 384. (Ed. Sartorelli & Johns). Berlin, Springer-Verlag.

TIDD, D.M. (1984). Antipurines. In: *Handbook of Experimental Pharmacology, Vol. 72: Antitumor Drug Resistance,* p. 445. (Ed. Fox & Fox). Berlin: Springer-Verlag.

TIDD, D.M. & DEDHAR, S. (1978). Specific and sensitive combined high performance liquid chromatographic-flow fluorometric assay for intracellular 6-thioguanine nucleotide metabolities of 6-mercaptopurine and 6-thioguanine. *J. Chromatogr.*, **145,** 237.

TIDD, D.M. & PATERSON, A.R.P. (1974*a*). Distinction between inhibition of purine nucleotide synthesis and the delayed cytotoxic reaction of 6-mercaptopurine. *Cancer Res.*, **34,** 733.

TIDD, D.M. & PATERSON, A.R.P. (1974*b*). A biochemical mechanism for the delayed cytotoxic reaction of 6-mercaptopurine. *Cancer Res.*, **34,** 738.

TIDD, D.M., GIBSON, I. & DEAN, P.D.G. (1982*a*). Partial circumvention of resistance to 6-mercaptopurine by acylated $P^1,P^2$-bis(6-mercaptopurine-9-β-D-ribofuranoside-5′) pyrophosphate derivatives. *Cancer Res.*, **42,** 3769.

TIDD, D.M., JOHNSTON, H.P. & GIBSON, I. (1982*b*). Effects of bis(6-mercaptopurine-9-β-D-ribofuranoside)-5′,5‴-phosphate and its butyryl derivative on mouse leukaemia L1210 and a 6-mercaptopurine-resistant subline in culture. *Biochem. Pharmacol.*, **31,** 2903.